# Continuous electrocardiogram changes preceding phenotypic expression for 8 years in an athlete with hypertrophic cardiomyopathy: a case report 

**DOI:** 10.1186/s13256-019-1998-7

**Published:** 2019-03-21

**Authors:** Dan Han, Yan Ji, Hui Tan

**Affiliations:** 1 Department of Cardiovascular Medicine, First Affiliated Hospital of Xi’an Jiaotong University, No. 277 Yanta West Road, Xi’an, Shaanxi 710061 People’s Republic of China; 2 Department of Rehabilitation Medicine, First Affiliated Hospital of Xi’an Jiaotong University, No. 277 Yanta West Road, Xi’an, Shaanxi 710061 People’s Republic of China

**Keywords:** Hypertrophic cardiomyopathy, ECG alterations, Abnormal Q waves, T-wave inversion, Athlete

## Abstract

**Background:**

Hypertrophic cardiomyopathy is one of the most common causes of sudden cardiac death in young athletes. Performing, comparing, and monitoring serial electrocardiograms over time can help to detect potential cardiovascular diseases and to prevent malignant cardiac events in these populations.

**Case presentation:**

A young Han Chinese male football player had abnormal electrocardiograms for 8 years without any subjective discomfort. Electrocardiograms revealed that T-wave inversions increased from 1 mm to a maximum of 5 mm on lead I and fluctuated around 5 mm on lead avL. Q-wave duration ranged from 40 ms to 60 ms, its depth increased to a maximum of 8 mm and was much greater than 40% of the R waves in depth in II, III, and avF leads. Echocardiography showed increasingly thickened interventricular septum from 10 mm to 13 mm, enlarged left atrium and ventricle, and reduced left ventricular ejection fraction. Coronary angiography showed no distinct stenosis. Emission computed tomography revealed mild myocardial ischemia of the left ventricular inferior wall. These unusual electrocardiogram manifestations were initially regarded as benign alterations of a highly trained athlete. Upon reviewing the clinical information and the newest criteria for electrocardiographic interpretation in athletes, hypertrophic cardiomyopathy was identified. The misreading of electrocardiograms is not uncommon, thus predisposing such patients to high susceptibility to exercise-induced sudden cardiac death.

**Conclusions:**

We propose that abnormal electrocardiogram findings reveal the initial expression of underlying cardiac diseases such as hypertrophic cardiomyopathy, preceding the symptoms and signs by many years. Accordingly, early detection and continuous surveillance are important for athletes with such electrocardiogram patterns, and improvement of physicians’ expertise is crucial.

## Introduction

Sudden cardiac death (SCD) is an important and emotionally charged public health issue [1, 2]. Hypertrophic cardiomyopathy (HCM) is the most frequent heart disease that causes SCD in the young population, especially in athletes during high-intensity training [3]. Athletes occasionally manifest with abnormal electrocardiogram (ECG) findings, many of which are usually considered as an inculpable presentation of cardiac structural remodeling in connection with intensive and systematic exercise [4]. However, in a minority of athletes, ECGs might be obviously abnormal and erratic, indicating the initial presence of a potential cardiac disease such as HCM, even when there is no visible structural heart condition [5]. Progressively abnormal ECG patterns may represent the initial, subtle expressions of cardiac diseases with long-lasting negative clinical outcomes [3]. In young athletes, the differential diagnosis between nonpathological alterations of cardiac structure related to training (generally termed “athlete’s heart”) and certain cardiac diseases with a risk of SCD remains a particularly significant clinical problem [6]. We report a case of a young athlete who was diagnosed as HCM without any discomfort or obvious alterations of visible heart morphology but manifested gradually aggravated abnormal Q waves and T-wave inversions (TWIs) on ECGs over the course of 8 years. This case report reveals that abnormal ECG alterations may precede the symptoms and signs of HCM for many years, and it further supports the fact that markedly aberrant cardiac electrical activity may represent the subtle expression of an underlying cardiac disorder that may not become evident until many years later. Accordingly, early detection and continuous surveillance are important for athletes with such ECG patterns, and improvements in physicians’ expertise are crucial.

## Case presentation

**Fig. 1:**
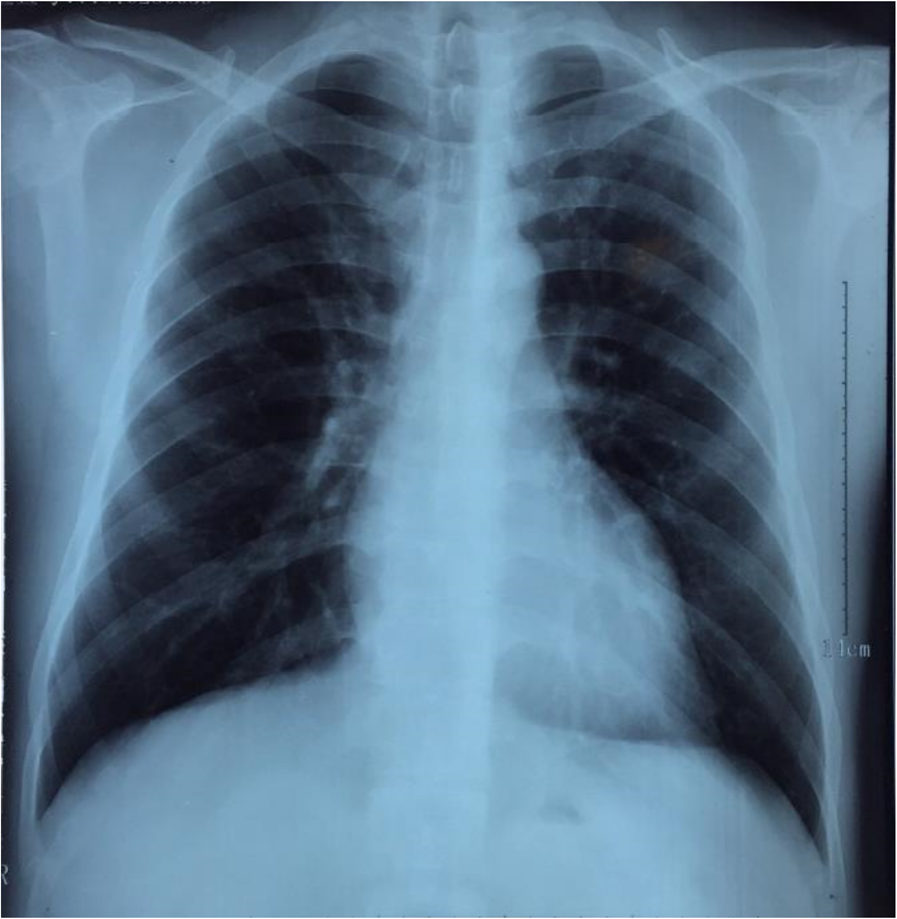
Chest radiography showed seemingly normal structures of heart and lungs

**Fig. 2:**
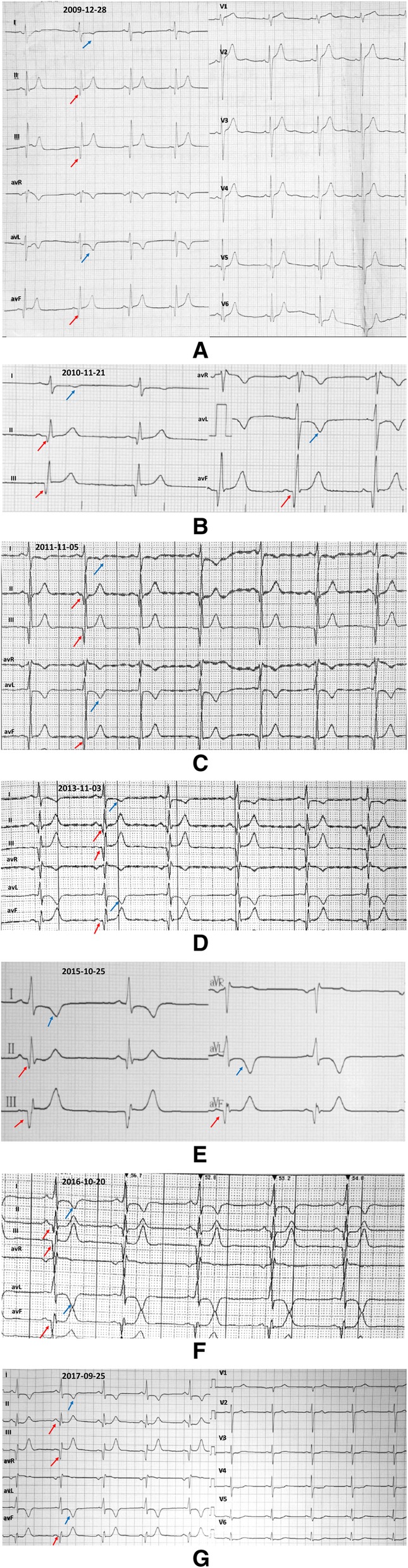
Electrocardiogram tracings recorded in 2009 (**a**), 2010 (**b**), 2011 (**c**), 2013 (**d**), 2015 (**e**), 2016 (**f**), and 2017 (**g**) indicated progressively deepened and widened Q waves (*red arrowheads*) and persistent T-wave inversion (*blue arrowheads*)

**Fig. 3:**
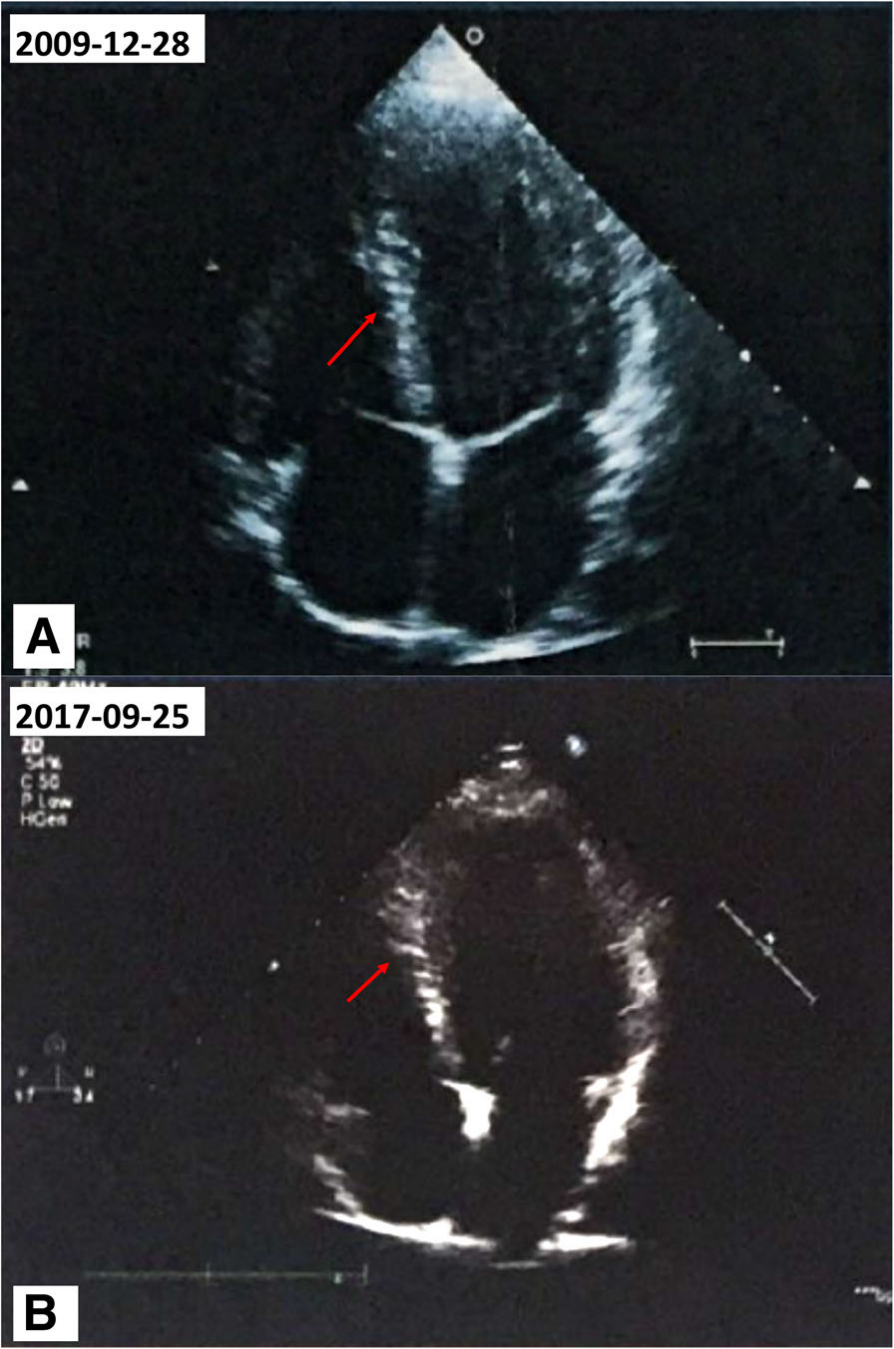
Echocardiography showed increasingly thickened interventricular septum from 10 mm (**a**) to 13 mm (**b**) (*red arrowheads*), enlarged left atrium and ventricle, and reduced left ventricular diastolic function

**Fig. 4:**
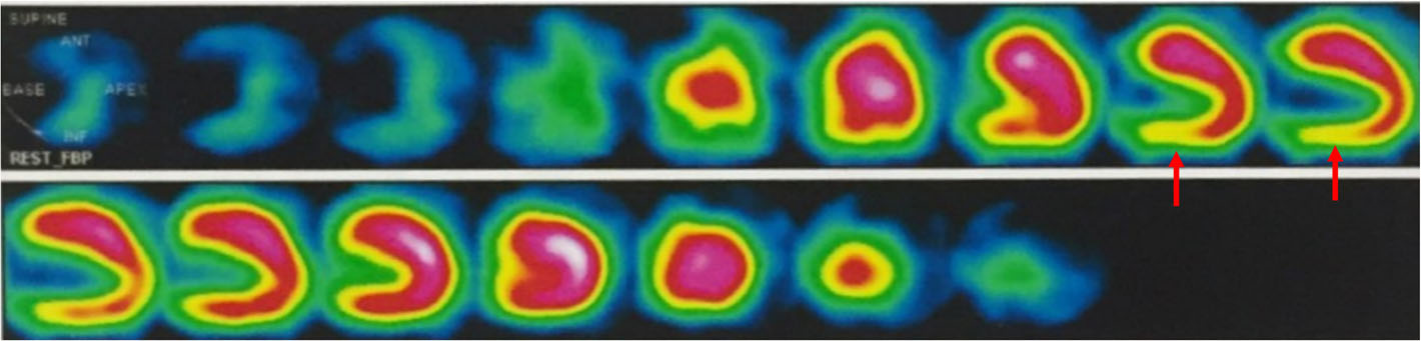
Emission computed tomography revealed mild myocardial ischemia of the left ventricular inferior wall (*red arrowheads*)

A 35-year-old Han Chinese male football player presented with abnormal ECGs for 8 years without any evident subjective discomfort. The patient recently complained about slight exertional dyspnea with reduced sport/physical tolerance and was admitted to our hospital. This patient had been employed as a professional football player from the age of 10 years and had been a physical education teacher from the age of 26 years. He was a longtime local resident and never went to any affected areas (areas with high prevalence of infectious diseases). He reported being formerly healthy without any medical histories or current comorbidities, and he reported taking no medications. He has smoked 20 cigarettes per day for 7 years and consumed alcohol for 10 years at 100 to 250 g per day. His parents were healthy, and his family history was unremarkable. His physical examination revealed no abnormal findings. His body temperature was 36.5 °C, blood pressure was 121/73 mmHg, respiratory rate was 18 breaths/min, pulse was 69/min, heart rate was 70 beats/min, and reflexes were normal. He had no pathology reflex, and his body mass index was 22.81 kg/m^2^. Laboratory evaluation revealed slightly elevated cardiac troponin T level of 0.017 ng/ml, N-terminal probrain natriuretic peptide level of 291.80 pg/ml, and C-reactive protein level of 0.40 mg/L. The patient’s blood lipid levels, liver function, and renal function were within the normal range with glutamic oxaloacetic transaminase level of 23 U/L, glutamic-pyruvic transaminase level of 31 U/L, alkaline phosphatase level of 84 U/L, total protein level of 67.9 g/L, albumin level of 41 g/L, globulin level of 26.9 g/L, total cholesterol level of 4.01 mmol/L, triglyceride level of 1.42 mmol/L, high-density lipoprotein level of 1.08 mmol/L, low-density lipoprotein level of 2.14 mmol/L, uric acid level of 353 μmol/L, epidermal growth factor receptor level of 105.48 ml/min/1.73 m^2^; serum K^+^ level of 4.05 mmol/L, serum Ca^2+^ level of 2.16 mmol/L, serum Mg^2+^ level of 0.88 mmol/L, and serum Na^+^ level of 141 mmol/L. The results of routine blood test and urinalysis were negative, thyroid function was normal, and microorganisms were not detected. Chest radiography showed an apparently normal morphology of the heart and lungs (Fig. 1). ECGs revealed progressively deepened and widened Q waves on the II, III, and avF leads and contiguous TWIs on the I and avL leads (Fig. 2, Table 1). Echocardiography revealed an increasingly thickened interventricular septum from 10 mm to 13 mm, an enlarged left atrium and ventricle, and a reduced left ventricular ejection fraction from 73% to 63% (Fig. 3). Coronary angiography (CAG) was performed and showed no distinct stenosis. Emission computed tomography (ECT) revealed mild myocardial ischemia of the left ventricular inferior wall (Fig. 4). All of these clinical tests supported the diagnosis of HCM, which became gradually evident with time. For further identification, we proposed other examination techniques for this patient, including cardiac magnetic resonance imaging (CMRI) to better evaluate the left ventricular wall thickness and to identify potential areas of myocardial fibrosis, Holter monitor recordings and an exercise test to evaluate possible “dynamic” changes of repolarization abnormalities, as well as genetic testing. However, the patient refused all of these suggestions and was discharged. In the subsequent follow-up visits at 1 month, 3 months, and 6 months after discharge, the patient showed poor compliance and was eventually lost to follow-up.

**Table 1:** Electrocardiogram measurement over 8 years

Date	TWIs depth (mm)		Q-wave duration (ms)			Q-wave depth (mm)			Q/R (%)		
	I	avL	II	III	avF	II	III	avF	II	III	avF
28/12/2009	1	5	40	40	40	4	6	4	40	43	33
21/11/2010	1	4	40	40	40	2	3.5	5.1	40	50	43
05/11/2011	2	4	40	40	40	6	8	5	60	67	42
03/11/2013	2.5	5	40	50	50	4	6	4	67	100	100
25/10/2015	5	5	40	50	40	3.5	5	4	58	125	200
20/10/2016	3.5	6	40	60	50	4	7	5	80	233	200
25/09/2017	3	5	40	50	40	3	5	4	60	100	133

*TWIs* T-wave inversions

## Discussion

Our patient manifested with abnormal ECGs, including progressively deepened and widened Q waves on the II, III, avF leads, and persistent TWIs on the I, avL leads. TWIs increased from 1 mm to a maximum of 5 mm on lead I and fluctuated approximately 5 mm on lead avL. The Q-wave duration ranged from 40 ms to 60 ms, and the depth increased to a maximum of 8 mm, which was much greater than 40% of the R-wave depth in the II, III, avF leads. Echocardiography showed an increasingly thickened interventricular septum from 10 mm to 13 mm, which is considered to be in the gray zone, and an enlarged left atrium and ventricle with reduced left ventricular ejection fraction from 73% to 63%. CAG revealed no distinct stenosis. ECT showed mild myocardial ischemia of the left ventricular inferior wall, which may present as pathological Q waves on ECGs. These alterations highly suggested the potential condition of HCM for this patient; thus, further examinations were recommended to confirm the diagnosis. Participation in competitive athletics was restricted as a timely prophylactic for longer survival. Pharmacological agents (for example, β-blockers) to control cardiac-related symptoms or ventricular arrhythmias and prophylactic implantable cardioverter-defibrillator were not applicable for this patient [7]. These ECG manifestations were initially regarded as benign alterations in this patient due to a shortage of physician expertise. This kind of phenomenon is not uncommon; therefore, an urgent need exists for physician education on modern ECG interpretation that distinguishes normal physiological adaptations in athletes from distinctly abnormal findings that are suggestive of underlying pathology.

The international consensus standards for ECG interpretation in athletes that suggest the potential for HCM include TWI ≥ 1 mm in depth in two or more contiguous leads, excluding III, aVR, and V1; and pathological Q waves ≥ 40 ms in duration and/or ≥ 3 mm in depth and/or Q/R ≥ 25% in at least two contiguous leads except for III and aVR [8, 9]. Pathological Q waves are the most common ECG findings associated with HCM and present in 32% to 42% of patients, especially those who exhibit an unusual pattern of ventricular hypertrophy [10]. The mechanism underlying HCM-related Q waves is an abnormal electrical activity of the hypertrophic ventricular septum [11]. Pathological studies revealed that abnormal Q waves that formed by the loss of electrical forces due to transmural myocardial fibrosis resembled those associated with myocardial infarction [12]. Another mechanism is that the resultant initial QRS vector alters its direction due to enhanced electrical forces of asymmetrical hypertrophy of the basal ventricular septum and/or basal left ventricular free wall, which is unopposed by apical electrical forces [13].

TWI indicates abnormal repolarization of the ventricular myocardium. In a cohort of young (aged < 35 years) asymptomatic patients with confirmed HCM, 62% exhibited abnormal TWI [10]. In patients with HCM without evident symptoms, the prevalence of abnormal TWI was reported as 80% [14]. More recently, the prevalence of abnormal TWI among athletes with newly diagnosed HCM was reportedly in excess of 90% [15]. TWI ≥ 1 mm on at least two contiguous leads except III, aVR, and V1 should be carefully assessed and should prompt further examinations, such as echocardiography, CMRI, an exercise ECG test, and a minimum of 24 h of ECG monitoring for underlying cardiac diseases [9].

## Conclusion

In our patient, abnormal ECG findings indicated that the initial expression of underlying cardiac diseases, such as HCM, preceded the signs and symptoms for many years. Accordingly, early detection and continuous surveillance are important for athletes who display such ECG patterns; moreover, improvements in physician expertise are crucial.
